# An Improvement in the Treatment of Epididymitis

**Published:** 1879-05-20

**Authors:** 


### *AN IMPROVEMENT IN THE TREATMENT OF EPIDIDYMITIS.*

In a paper contributed to the Allgemeine Wiener Med. Zeitung, Prof. Zeissl, of Vienna, gives an account of the results of the trials he has made of the treatment of this affection pursued by Mr. Horand, of Lyons.

As to the statistics of the affection, he observes that of 2055 men suffering from gonorrhœa who have come under his care at the hospital during the last eight years, 696 have been attacked by epididymitis, 368 suffering from it on the right side, 302 on the left side, and 26 on both sides. The mode of treating the affection during the last ten years has been the antiphlogistic, consisting in the application of leeches and cold compress along the course of the cord to the perineum, and the employment of purgatives. Not only has the diet been more or less limited, but the patients have been confined to bed. The scrotum, exhibiting more or less erysipelatous redness with tumefaction, has been kept up constantly by means of a cushion, or raised upon the thighs by a cloth of a hand's breadth passed under it. It is also kept surrounded by a wet compress, over which is laid a small light bladder of ice. In many cases these means suffice in three or four weeks to relieve the tumefaction of the vas deferens of the epididymis, and the inflammatory conditions of the skin, of the scrotum and the subscrotal tissue, and to allow of the restriction on diet being removed. Only in exceptional cases, and when the epididymitis has assumed the chronic form, may practitioners apply the unguentum hydrargyri cinereum in moderate quantities to the affected side of the scrotum, continuing the local employment of cold. Formerly many had great dread of the application of cold, under the idea that revulsion of the diseased process to the lungs might be induced, erroneously supposing that some fatal cases of hæmoptysis which have occurred were due to this application of cold. When the pain has been excessive, Professor Zeissl has applied belladonna ointment (five parts of the extract of belladonna to fifty of simple ointment) spread upon linen or leather; or has had a portion the size of a nut rubbed into the diseased side of the scrotum, continuing the cold applications, and securing sufficient action of the bowels.

Although by following this method a very great number of cures of this affection were obtained, yet, owing to several inconveniences it gave rise to, other means of treatment were sought for. One of the earliest of this was that of Frick, which consisted in submitting the testis to a regulated compression by means of strips of sticking plaster. This, however, could only be applied after the excessive sensitiveness of the parts had subsided. Even then it required the greatest care, and Prof. Zeissl has repeatedly known gangrene of the integument of the scrotum follow its employment. Also, in other cases in which the compression has been too hastily applied, he has known wasting of the testis to follow. Simplification of the treatment of gonorrhœal epididymitis therefore continued a desideratum; and that the more so because the painful nature of the affection kept the patient entirely away from his employment, while the nature of the affection becoming known often operated mischievously against his interests. It therefore became very desirable to find some means which might at least partly relieve the pain and enable the patient to resume his employment. This seems to be the merit of Langlebert's method as described by Horand, surgeon to the Antiquaille at Lyons, in a work bearing the title "Du Traitement de la Tumeur Blenorrhagique des Bourses par le Pansement Ouato-caoutchoute de Langlebert." In this accounts are given of 200 cases successfully treated; and the present paper furnishes the results of Prof. Zeissl's trials with the apparatus in question. This consists of three parts—a layer of wadding of a sufficient degree of thickness, a square piece of caoutchouc cloth, and a suspensory. This last, triangular in shape, and slightly concave, has a hole at its upper edge through which the penis is passed. To its two upper corners are attached two long bands which serve to confine it around the abdomen; and the lower is also attached to two bands which surround the thighs, these last being connected with and fastened to the bandage that goes around the abdomen. The patient lying in the horizontal posture, raises the scrotum well enveloped by wadding as high as possible upon the symphysis pubis; and then the square piece of caoutchouc cloth is applied with its shining side towards the wadding, a circular hole having been made in its upper part for the passage of the penis. The suspensory is then put on, and firmly secured to the abdominal band. By this means the scrotum is kept up almost level with the upper edge of the pubes. Horand does not undo the bandage until the end of a week, and if he finds the swelling has not yet disappeared he continues the procedure or applies a resolvent ointment or plaster.

Prof. Zeissl strongly recommends the procedure, having treated by it in a short time fifty cases, either in private or hospital practice, and always with most excellent results. He relates a case to which he was called, in which the patient, who had passed five nights without sleep, was suffering fearful pains, every motion and the slightest contact with the testis giving him agony. The Langlebert apparatus was applied, and the patient desired to rise from his bed. This, from the intense pain he had suffered before trying to do so, he did with great hesitation, but found that he could get up without the slightest suffering, and was able even to walk to and fro in the room. Prof. Zeissl states that he could cite several similar cases, and up to the present time he has never had, in any of the so treated cases, to prescribe anodyne ointments, or to puncture an acute hydrocele produced by the epididymitis. In most of the cases the pain ceased immediately on applying the bandage, or at the very least was so diminished as to allow the patient to pursue his employment. The practice is so simple and so cheap that it is especially suited for hospital practice, the bandage only having to be applied, and all the attendance formerly required, as the application of ice, etc., dispensed with. The suspensory used is far superior to that now commonly employed, there being none of the mischievous pressure from the elastic; while the scrotum is almost completely immobilized, and an exact application is secured by means of the wadding. A very moderate compression is exerted, and an abundant transpiration takes place at the surface of the scrotum. In all these fifty cases the patients were able to walk about the room, and most of them to follow their employments.—*Amer. Journal of Medical Sciences.*

